# Oral doxycycline for the prevention of postoperative trachomatous trichiasis in Ethiopia: a randomised, double-blind, placebo-controlled trial

**DOI:** 10.1016/S2214-109X(18)30111-6

**Published:** 2018-04-10

**Authors:** Esmael Habtamu, Tariku Wondie, Sintayehu Aweke, Zerihun Tadesse, Mulat Zerihun, Bizuayehu Gashaw, Chrissy H Roberts, Amir Bedri Kello, David C W Mabey, Saul N Rajak, E Kelly Callahan, David Macleod, Helen A Weiss, Matthew J Burton

**Affiliations:** a Medical Research Council Tropical Epidemiology Group, London School of Hygiene & Tropical Medicine, London, UK; b International Centre for Eye Health, London School of Hygiene & Tropical Medicine, London, UK; c Clinical Research Department, London School of Hygiene & Tropical Medicine, London, UK; d The Carter Center, Addis Ababa, Ethiopia; e Amhara Regional Health Bureau, Bahirdar, Ethiopia; f Light for the World, Addis Ababa, Ethiopia; g The Carter Center, Atlanta, GA, USA

## Abstract

**Background:**

Trachomatous trichiasis is treated surgically to prevent sight loss. Unfavourable surgical outcomes remain a major challenge. We investigated the hypothesis that doxycycline might reduce the risk of postoperative trichiasis following surgery in patients with trachomatous trichiasis through anti-matrix metalloproteinase and anti-inflammatory activity.

**Methods:**

In this randomised, double-blind, placebo-controlled trial, adults (aged >18 years) with upper lid trachomatous trichiasis in association with tarsal conjunctive scarring were recruited through community-based screening and surgical outreach campaigns in Ethiopia. Individuals who had previously had eyelid surgery were excluded. Participants were randomly assigned (1:1), with random block sizes of four or six, to receive oral doxycycline (100 mg once a day) or placebo for 28 days immediately after trichiasis surgery. Randomisation was stratified by surgeon. Patients, investigators, surgeons, and all other study team members were masked to study group allocation and treatment. Participants were examined at 10 days, and 1, 6, and 12 months after surgery. The primary outcome was the cumulative proportion of individuals who developed postoperative trichiasis by 12 months. Primary analyses were done in all participants who attended at least one of the four follow-up assessments. Safety analyses were done in all participants who attended either the 10 day or 1 month follow-up assessments. This trial is registered with the Pan African Clinical Trials Registry, number PACTR201512001370307.

**Findings:**

Between Dec 21, 2015, and April 6, 2016, 1000 patients with trichiasis were enrolled and randomly assigned to treatment (499 patients to doxycycline, 501 patients to placebo). All but one participant attended at least one follow-up assessment. Thus, 999 participants were assessed for the primary outcome: 498 in the doxycycline group and 501 in the placebo group. By month 12, 58 (12%) of 498 patients in the doxycycline group and 62 (12%) of 501 patients in the placebo group had developed postoperative trichiasis (adjusted odds ratio 0·91, 95% CI 0·61 to 1·34, p=0·63), with a risk difference of −0·5% (–4·5% to 3·5%). Significantly more patients in the doxycycline group had an adverse event than in the placebo group (18 [4%] of 498 *vs* six [1%] of 501; odds ratio 3·09, 95% CI 1·21–7·84; p=0·02). The most frequent adverse events in the doxycycline group were gastritis symptoms (n=9), constipation (n=4), and diarrhoea (n=4).

**Interpretation:**

Doxycycline did not reduce the risk of postoperative trichiasis and is therefore not indicated for the improvement of outcomes following trachomatous trichiasis surgery. Surgical programmes should continue to make efforts to strengthen surgical training and supervision to improve outcomes.

**Funding:**

The Wellcome Trust.

## Introduction

Trachoma is a leading infectious cause of blindness worldwide. The neglected tropical disease is caused by *Chlamydia trachomatis*. Recurrent *C trachomatis* infections cause chronic inflammation and conjunctival scarring. The eyelids turn inwards and eyelashes scratch the surface of the eye (trachomatous trichiasis), leading to sight loss due to corneal opacification. Globally, an estimated 1·9 million people are visually impaired, of whom 0·45 million have irreversible blindness caused by trachoma.^1^ About 3·2 million people are estimated to have untreated trachomatous trichiasis and are thus at considerable risk of sight loss.^2^ The WHO Alliance for the Global Elimination of Trachoma by 2020^3^ recommends the SAFE Strategy for trachoma control,^4^ which consists of surgery for trachomatous trichiasis, annual mass antibiotic treatment with azithromycin, and facial cleanliness and environmental improvement strategies to suppress transmission.

Unfavourable outcomes after trichiasis surgery are a considerable concern for trachoma control programmes, and are reported to occur in around a third of patients. The most common adverse postoperative outcomes include postoperative trichiasis (about 20% of patients), eyelid contour abnormalities (around 10% of patients), and conjunctival granulomas (about 5% of patients).^5^, ^6^, ^7^, ^8^, ^9^ Postoperative trichiasis can lead to vision loss and pain and has the potential to undermine elimination efforts. On the basis of current clinical practice more than 54 000 patients who have surgery for trichiasis develop trachomatous trichiasis annually.^1^ The leading factors reported to contribute to the development of postoperative trichiasis include type of surgical procedure, surgeon skill, severity of preoperative disease, patient age, and ongoing conjunctival inflammation and scarring.^5^, ^6^, ^7^, ^8^, ^10^, ^11^

**Research in context**

**Evidence before this study**

Doxycycline has been widely used in various clinical situations for its putative anti-inflammatory and anti-scarring properties. In the first weeks after trachomatous trichiasis surgery, progressive conjunctival inflammation and contractile scarring mediated by matrix metalloproteinase and inflammatory cytokine activity might lead to unfavourable outcomes. On Sept 13, 2017, we updated a search done for a published Cochrane Collaboration systematic review of the management of trachomatous trichiasis (Burton and colleagues, 2015). We searched CENTRAL, Ovid MEDLINE, Embase, the International Clinical Trials Registry Platform, ClinicalTrials.gov, and WHO International Clinical Trials Registry Platform using the search terms “trachoma” and “trichiasis”. Full search methods for each database are available in the Cochrane review's appendix. We found no studies examining the effect of doxycycline on trichiasis surgery outcomes.

**Added value of this study**

To the best of our knowledge this is the first trial investigating the use of doxycycline to improve outcomes following trichiasis surgery. Our trial was done to investigate whether oral doxycycline might improve trachomatous trichiasis surgery outcomes by suppressing progressive inflammation and contractile scarring during the surgical wound healing process. The results show that oral doxycycline does not improve surgical outcomes following trichiasis surgery, and does not affect the extent of postoperative conjunctival scarring or conjunctival inflammation, whether this is related to the initial wound healing process or longer-term chronic progression.

**Implications of all the available evidence**

This study provides evidence that oral doxycycline does not improve outcomes in trachomatous trichiasis surgery, and therefore should not be indicated for this purpose. Consistent with previous studies, our study showed that other factors, such as preoperative disease severity, surgeon skill, and patient age are important determinants of outcome, suggesting that trachoma control programmes should focus on addressing these factors and continue to make efforts to improve surgical training and supervision. Further studies are required to investigate the association between postoperative scarring and trichiasis surgery outcomes.

Conjunctival inflammation is frequently observed in people with trichiasis.^10^, ^12^, ^13^, ^14^, ^15^, ^16^, ^17^, ^18^ The drivers of this persistent inflammation in individuals with scarring and trichiasis have not been fully elucidated; however, it is rarely associated with *C trachomatis* infection.^16^, ^19^ Chronic conjunctival inflammation is associated with progressive conjunctival scarring.^20^ Studies^16^ in Ethiopia and Tanzania found that chronic conjunctival inflammation in trachomatous scarring was associated with the expression of proinflammatory cytokines. Conjunctival scarring was also associated with increased conjunctival expression of matrix metalloproteinases (MMPs), such as MMP-7, MMP-9, and MMP-12.^21^ MMPs degrade the extracellular matrix, promote inflammatory cell infiltration, and facilitate tissue remodelling and scar formation. In our previous studies,^22^, ^23^ following trichiasis surgery, patients had altered expression of several pro-inflammatory cytokines and MMPs, which could favour postoperative scarring. In other types of conjunctival surgery (eg, trabeculectomy for glaucoma), poor outcomes occur because of excessive inflammation and fibrosis during initial wound healing, which is mediated by fibroblast and MMP activity. Similar alterations in wound healing processes are likely to occur following trichiasis surgery and might contribute to the development of postoperative trichiasis and eyelid contour abnormalities.

To reduce early postoperative trachomatous trichiasis due to contractile scarring it might be beneficial to inhibit MMP and inflammatory cytokine activity. However, at present, interventions used in other types of ophthalmic surgery (ie, mitomycin and fluorouracil) would not be suitable for patients who have surgery for trachomatous trichiasis because of their toxicity, specific handling requirements, and high cost. Doxycycline is a widely available, broad-spectrum, tetracycline-based antibiotic that is effective against *C trachomatis,* and also has anti-inflammatory and anti-MMP properties.^24^ Doxycycline inhibits MMP activity via several mechanisms: reduced MMP expression, blocking pro-MMP activation, and direct inhibition by binding Zn^2+^ and Ca^2+^ ions at the catalytic site.^24^ An in-vitro study^25^ examined collagen matrix contraction in tarsal conjunctival fibroblasts cultured from biopsy samples of individuals who had trichiasis surgery. The expression of MMP-1, MMP-2, MMP-7, MMP-9, and MMP-12 was significantly higher during the contraction assay than at baseline. The addition of doxycycline reduced collagen gel contraction by up to 75%, with no evidence of toxicity. MMP production was significantly reduced by the doxycycline treatment. An ex-vivo study^26^ investigating the inhibitory effects of doxycycline on MMP-9 in brain arteriovenous malformations showed that doxycycline significantly decreases MMP-9 concentrations.

Doxycycline has been widely used in various clinical situations because of its putative anti-inflammatory and anti-scarring properties, mediated via the inhibition of MMPs and inflammatory cytokines, including periodontitis, pulmonary fibrosis, and vascular disorders, and in ophthalmology for treating ocular surface disorders, such as ocular rosacea, severe microbial keratitis, alkali injuries, and recurrent corneal erosion syndrome.^27^, ^28^, ^29^, ^30^, ^31^, ^32^, ^33^, ^34^ Doxycycline is considered a safe drug, and has been tested in a community-wide treatment study of onchocerciasis,^35^, ^36^ in which patient adherence was high (97%) and no serious side-effects were reported.

In view of the unfavourable outcomes following surgery in patients with trachomatous trichiasis and the anti-MMP and anti-inflammatory properties of doxycyline, we hypothesised that doxycycline might improve surgical outcomes. The aim of our trial was to determine whether oral doxycycline can reduce the risk of postoperative trichiasis and eyelid contour abnormalities following surgery in patients with trachomatous trichiasis.

## Methods

### Study design and participants

We did a double-blind, randomised, placebo-controlled trial in Ethiopia. Participants were mainly recruited through community-based screening by a mobile eye care team and by organising community-based surgical outreach campaigns in various health facilities across five districts of the West Gojam Zone in the Amhara Region.

Eligible participants (aged >18 years) had upper lid trachomatous trichiasis (one or more eyelashes touching the eye or evidence of epilation) with tarsal conjunctival scarring. We excluded people who had previously had eyelid surgery, individuals with trichiasis resulting from other eyelid pathologies, and people with contraindications to doxycycline use, including renal or liver impairment, myasthenia gravis, and people taking warfarin, sulphonylureas, or doxycycline. Individuals with hypertension (blood pressure >170/110 mm Hg), known allergy to doxycycline or tetracycline, and pregnant or lactating women were also excluded. Written informed consent was obtained from all participants in Amharic before enrolment. Illiterate participants were read the information sheet and consent form, and their consent was recorded by thumbprint in the presence of a witness.

The study was approved by the Ethiopian National Health Research Ethics Review Committee, the London School of Hygiene & Tropical Medicine Ethics Committee, Emory University Institutional Review Board, and the Ethiopia Food, Medicine and Healthcare Administration and Controls Authority, and was done in accordance with the Declaration of Helsinki and International Conference on Harmonisation–Good Clinical Practice guidelines.

### Randomisation and masking

Participants were randomly assigned (1:1) to receive either doxycycline or placebo once daily for 28 days, with a random block size of four or six. Randomisation was stratified by surgeon because of potential intersurgeon variability. An independent statistician was responsible for computerised sequence generation. The allocation sequences for each surgeon were concealed in sequentially numbered, sealed, opaque envelopes, which were prepared by a person independent of all other aspects of the trial.

After meeting inclusion criteria and completing baseline assessments, participants were allocated to the next available surgeon. On most recruitment days, two surgeons operated simultaneously. Immediately after surgery, patients were brought to the randomisation area by their surgeon and were assigned to a study group by a pharmacist who opened the next envelope in that surgeon's sequence to determine allocation.

The patient, the two outcome assessors (EH and SA), the pharmacist, the surgeons, and all other study team members were masked to study group allocations and treatment. The doxycycline and placebo were produced as encapsulated medication by East African Pharmaceuticals (Addis Ababa, Ethiopia). The medication boxes, blister strips, and doxycycline and placebo capsules were identical in appearance.

### Procedures

Participants' demographic characteristics were recorded at the preoperative assessment before randomisation. Visual acuity at 2 m was assessed according to the logarithm of the minimum angle of resolution (logMAR) using PeekAcuity software (Peek Vision, London, UK) on a smartphone in a dark room.^37^ For visual acuities of counting fingers or less, logMAR values were assigned as follows: counting fingers, 2·0; hand movements, 2·5; perception of light, 3·0; and no perception of light, 3·5.^17^

Eyes were examined by a single examiner (EH) using 2·5 × binocular loupes and a torch, and graded using a Detailed WHO (Follicles, Papillae, Cicatricae) Grading System.^38^ Eyelashes touching the eye were counted and subdivided by the part of the eye contacted (cornea, lateral, or medial conjunctiva). Trichiasis subtypes were defined as metaplastic, misdirected, and entropic.^39^ Clinical evidence of epilation was identified by broken or newly growing eyelashes, or areas of the eyelid without lashes. Entropion of the upper lid was graded according to the degree of eyelid margin inward rotation.^39^ Corneal scarring was graded using detailed criteria,^40^ based on an expansion of the WHO (Follicles, Papillae, Cicatricae) Grading System. Four standardised high-resolution digital photographs of trichiasis (in straight gaze and up gaze), the cornea, and tarsal conjunctiva were taken, using a Nikon D90 digital SLR camera with 105 mm macro lens and R1C1 flash units (Nikon, Tokyo, Japan).^41^

Before recruitment, we selected ten experienced trichiasis nurse-surgeons, who did posterior lamellar tarsal rotation (PLTR) surgery regularly within the Amhara Region trachoma control programme. The nurse-surgeons had refresher training, which was standardised by an experienced ophthalmologist using the WHO manual assessment procedure. The best six surgeons were chosen to do the surgery in the trial. Participants were treated surgically using PLTR, following the procedures described in the WHO Trichiasis Surgery for Trachoma manual.^42^ Postoperatively, operated eyes were padded for 1 day and tetracycline eye ointment 1% was self-administered twice a day for 2 weeks.

Immediately after trichiasis surgery, patients were randomly assigned to receive oral doxycycline 100 mg or placebo 100 mg for 28 days. Participants were examined at 10 days, 1 month, 6 months, and 12 months after surgery. On day 10, participants were examined for postoperative trichiasis and surgical complications before suture removal. At 1 month, 6 month, and 12 month follow-up assessments, participants were re-examined (by SA at 1 month and 6 months and EH at 12 months) and photographed following the same procedures used at baseline. The examiners (EH and SA) were standardised and had very strong agreement for the primary outcome in grading validation studies (κ=0·92). On the basis of severity, postoperative trichiasis was categorised as minor trichiasis (less than six eyelashes touching the eye, or evidence of epilation in less than a third of the eyelid margin) and major trichiasis (six or more eyelashes touching the eye, or evidence of epilation in a third or more of the eyelid margin). Individuals who had postoperative trichiasis at any follow-up assessment were offered epilation or repeat surgery by the most experienced surgeon in the area on the basis of severity using previously defined criteria.^43^ Grading of entropion correction, granuloma, and eyelid contour abnormalities was done according to previously described criteria.^7^ Participants with other ophthalmic pathologies were referred to the nearest eye unit.

At day 10 and 1 month follow-up assessments, participants were interviewed about drug adherence and adverse events. Drug adherence was assessed by asking participants whether they had stopped taking the treatment and by counting the number of capsules remaining. If the participant did not bring the medication box with them to the follow-up, a member of the study team visited the participant's home to count the number of capsules remaining.

### Outcomes

The primary outcome was the cumulative proportion of individuals who developed postoperative trichiasis by 12 months (ie, patients diagnosed with postoperative trichiasis at any one of the four follow up visits within 12 months of surgery). Postoperative trichiasis was defined as one or more eyelashes touching the eye or clinical evidence of epilation, or a history of repeat trichiasis surgery by 12 months.

A-priori defined secondary outcomes were: postoperative trichiasis at 1 month, 6 months, and 12 months; postoperative trichiasis difference by surgeon; postoperative trichiasis difference by baseline disease severity; number, type, and location of postoperative eyelashes at 12 months; eyelid contour abnormalities at 1 month, 6 months, and 12 months; corneal opacity and vision changes at 12 months; postoperative surgical complications (infection and granulomas); and patient reported adverse events in the first month after surgery.

### Statistical analysis

A previous randomised trial in Ethiopia^17^ of patients who had PLTR surgery reported a 1-year postoperative trichiasis risk of 18%. We estimated that a sample of 836 participants would be needed to detect a clinically significant difference in postoperative trichiasis (18% *vs* 10%) with 90% power. We aimed to recruit 1000 participants (500 per group), allowing for about 15% loss to follow-up.

For participants who had bilateral surgery, we randomly designated one eye as the study eye for the analysis. Analyses were by modified intention to treat, with primary outcome data analysed for all participants seen at any of the four follow-up assessments. Participants who did not attend at least one of the four follow-up assessments were excluded from the analysis. The effect of the intervention on the primary and binary secondary outcomes was analysed using logistic regression to estimate the odds ratio (OR) and 95% CI. Intervention effects on ordered categorical secondary outcomes (ie, changes in visual acuity and corneal opacity) were assessed using ordinal logistic regression. Effects on categorical secondary outcomes (ie, type and location of postoperative trichiasis eyelashes, eyelid contour abnormalities, and entropion correction) were assessed using multinomial logistic regression to estimate relative risk ratio and 95% CI. Negative binomial regression was used to analyse the difference in the number of postoperative lashes touching the eye between the two groups. A non-prespecified sign test was used to analyse the clinical significance of eyelid contour abnormalities improvement between 1 month and 6 months, 1 month and 12 months, and 6 months and 12 months in all participants. A multivariable logistic regression model was used to identify potential explanatory factors for postoperative trichiasis by 1 year, granuloma by 1 year, and eyelid contour abnormalities at 1 year, in all study participants regardless of intervention allocation. All variables with a p value of less than 0·05 in the univariable model were included in the multivariable analysis and a likelihood ratio test was used to establish the variables for inclusion in the final multivariable logistic regression model at a p value of less than 0·20.

All comparisons between the two treatment groups were controlled for surgeon in the model to account for the stratified randomisation, and for preoperative disease severity. The risk difference in the primary outcome between the two groups was estimated. Effect modification between treatment group and a-priori defined factors such as surgeon, preoperative trichiasis severity, papillary inflammation, age, and sex was investigated by including interaction terms in the model. Stratum specific ORs for the primary outcome were calculated with evidence of interaction with the intervention. A per-protocol analysis of the primary outcome was done using logistic regression for all participants who adhered to treatment. Participants were considered to have adhered to treatment if they reported at the 1 month follow-up that they had taken the allocated treatment without stopping for more than 1 day.

An independent data and safety monitoring committee oversaw the trial. Data were double-entered into Microsoft Access 2007 (Microsoft, Redmond, WA, USA) and transferred to Stata 11 (StataCorp, College Station, TX, USA) for analysis. This study is registered with the Pan African Clinical Trials Registry, number PACTR201512001370307.

### Role of the funding source

The funder of the study had no role in study design, data collection, data analysis, data interpretation, or writing of the report. The corresponding author had full access to all the data in the study and had final responsibility for the decision to submit for publication.

## Results

Between Dec 21, 2015, and April 6, 2016, 3593 individuals were assessed for eligibility, of whom 1722 (48%) had trichiasis, 1422 (40%) had another ocular problem, and 449 (12%) had no ocular problems. Of the 1722 individuals with trichiasis, 707 (41%) were excluded: 344 (49%) had postoperative trichiasis, 175 (25%) had trichiasis without clear tarsal conjunctival scarring, 111 (16%) were lactating, 19 (3%) were pregnant, 16 (2%) had high blood pressure, 12 (2%) had mental health or hearing problems, nine (1%) were younger than 18 years, seven (1%) had kidney or liver problems, six (1%) were unable to attend follow-up, six (1%) were taking other multiple drugs, one (<1%) was taking doxycycline, and one (<1%) was seen after recruitment had been completed. Of the 1015 eligible participants, 15 (1%) declined surgery. Thus, 1000 individuals with trichiasis were enrolled, and randomly assigned to receive doxycycline (499 patients) or placebo (501 patients; figure).

**Figure:**
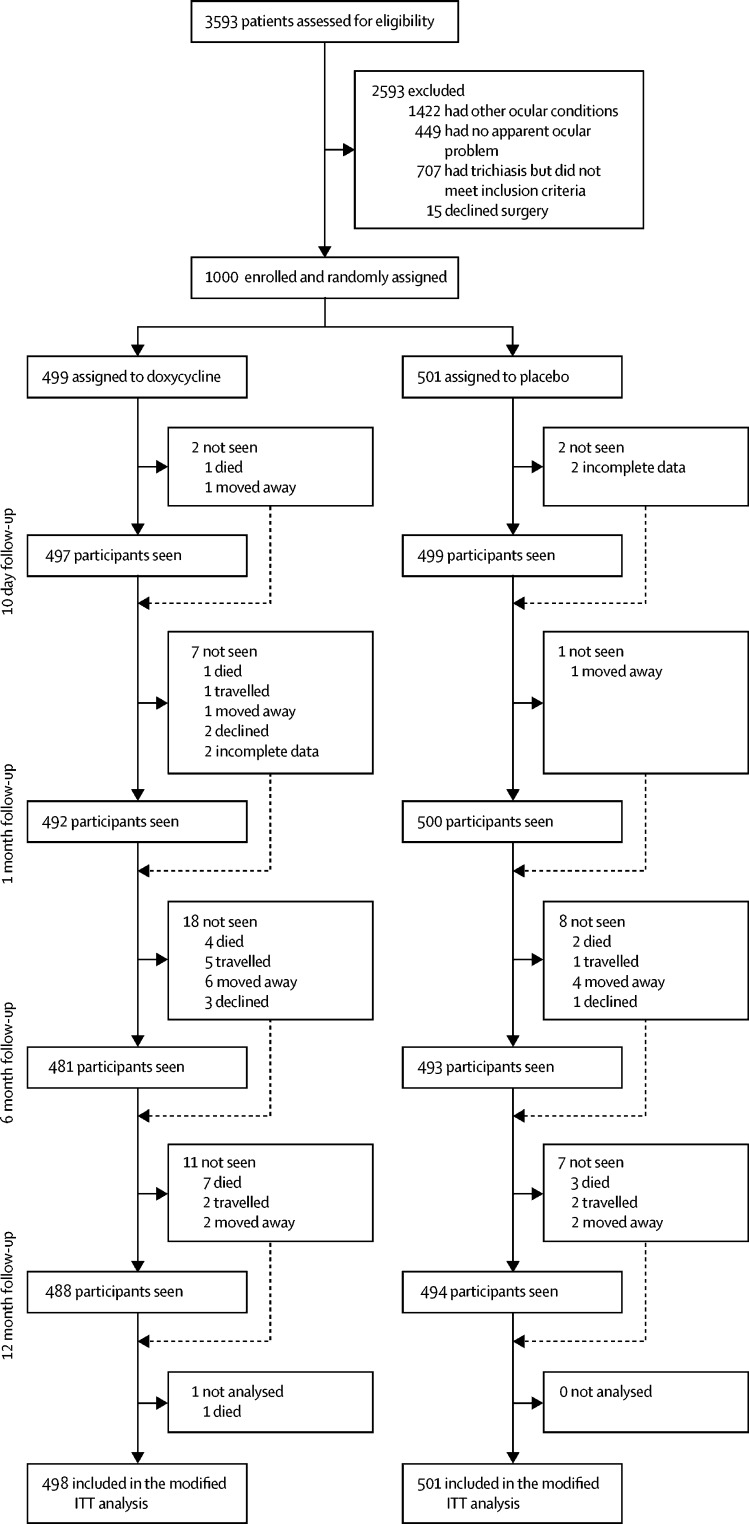
Trial profile ITT=intention-to-treat.

All but one participant in the doxycycline group attended at least one follow-up assessment. Thus, primary outcome data were available for 999 (100%) of 1000 trial participants: 498 in the doxycycline group and 501 in the placebo group. Of the 992 participants seen at the 1 month follow-up (492 [99%] of 498 in the doxycycline group; 500 [100%] of 501 in the placebo group), 815 (82%) participants had adhered to study treatment: 392 (48%) in the doxycycline group, and 423 (52%) in the placebo group (OR 0·71, 95% CI 0·51–0·99, p=0·04). Adherence was similar between sexes (228 [80%] of 284 men *vs* 587 [83%] of 708 women). However, older individuals (aged ≥60 years) were less likely to adhere to the study drug than younger individuals (OR 0·77, 95% CI 0·68–0·87, p<0·0001).

Baseline demographic and clinical characteristics were balanced between the two groups, with the exception of preoperative trichiasis severity (table 1). The mean age was 51·0 years (SD 14·1), and most participants were women (701 [70%]), and illiterate (881 [88%]). Clinical features, including visual acuity, corneal opacity, tarsal conjunctival scarring and inflammation, and entropion grade were similar across the two groups. The number and location of eyelashes touching the eye were balanced between the two groups. However, the proportion of participants with major trichiasis was higher in the doxycycline group than the placebo group (255 [51%] *vs* 222 [44%]) when extent of epilation was included in the trichiasis severity definition. 272 (54%) of 501 participants in the placebo group and 274 (55%) of 498 participants in the doxycline group had evidence of epilation. Of the 546 participants with epilation, 327 (60%) had epilation in less than a third of the eyelid (165 [33%] in the placebo group *vs* 162 [32%] in the doxycycline group), and 219 (40%) had epilation in a third or more of the eyelid margin (107 [21%] *vs* 112 [22%]). 25 (5%) of 501 participants in the placebo group and 34 (7%) of 499 participants in the doxycycline group successfully epilated (no lashes touching).

**Table 1:** Baseline and 12 month characteristics of participants

		**Baseline**		**12 months**	
		Placebo(n=501)	Doxycycline (n=499)	Placebo (n=494)	Doxycycline (n=488)
Sex					
	Women	365 (73%)	345 (69%)	..	..
	Men	136 (27%)	154 (31%)	..	..
Age, years		49·9 (14·0)	52·1 (14·3)	..	..
Illiterate		433 (86%)	449 (90%)	..	..
Best corrected logMAR visual acuity in study eye					
	−0·1 to 0·3	139 (28%)	134 (27%)	192 (39%)	167 (34%)
	0·3 to 0·7	231 (46%)	243 (49%)	212 (43%)	210 (43%)
	0·7 to 1·1	101 (20%)	87 (17%)	67 (14%)	88 (18%)
	1·1 to 2·0	7 (1%)	9 (2%)	3 (1%)	6 (1%)
	CF/HM/PL	22 (4%)	25 (5%)	18 (4%)	16 (3%)
	NPL	1 (<1%)	1 (<1%)	1 (<1%)	1 (<1%)
	Not possible to measure	..	..	1 (<1%)	..
Entropion grade					
	0	1 (<1%)	0	447 (90%)	436 (89%)
	1	21 (4%)	27 (5%)	33 (7%)	43 (9%)
	2	415 (83%)	408 (82%)	14 (3%)	9 (2%)
	3	57 (11%)	56 (11%)	0	0
	4	7 (1%)	8 (2%)	0	0
Trichiasis (number of eyelashes)					
	No trichiasis	..	..	450 (90%)	441 (90%)
	None (epilating)	25 (5%)	34 (7%)	4 (1%)	3 (1%)
	1–5	292 (58%)	268 (54%)	39 (8%)	43 (9%)
	6–9	96 (19%)	107 (21%)	0	1 (<1%)
	10–19	68 (14%)	62 (12%)	0	0
	≥20	20 (4%)	28 (6%)	1 (<1%)	0
	Mean (SD)*	6·3 (6·8)	6·9 (8·0)	2·5 (3·9)	1·9 (1·4)
Epilation					
	Not epilating	229 (46%)	225 (45%)	484 (97%)	483 (99%)
	Epilation in less than a third of eyelid margin	165 (33%)	162 (32%)	7 (1%)	4 (1%)
	Epilation in a third or more of the eyelid margin	107 (21%)	112 (22%)	3 (1%)	1 (<1%)
Trichiasis severity					
	Minor	279 (56%)	244 (49%)	43 (9%)	45 (9%)
	Major	222 (44%)	255 (51%)	1 (<1%)	2 (<1%)
Lash location					
	None (epilating)	25 (5%)	34 (7%)	4 (1%)	3 (1%)
	Corneal only	406 (81%)	395 (79%)	28 (6%)	31 (6%)
	Corneal and peripheral	62 (12%)	68 (14%)	5 (1%)	3 (1%)
	Peripheral only	8 (2%)	2 (<1%)	7 (1%)	10 (2%)
Corneal opacity					
	None (CC0)	88 (18%)	94 (19%)	153 (31%)	163 (33%)
	Peripheral (CC1)	174 (35%)	164 (33%)	115 (23%)	118 (24%)
	Off centre faint (CC2a)	89 (18%)	112 (22%)	75 (15%)	77 (16%)
	Off centre dense (CC2b)	10 (2%)	14 (3%)	9 (2%)	6 (1%)
	Central faint (CC2c)	128 (26%)	91 (18%)	127 (26%)	108 (22%)
	Central dense (CC2d)	12 (2%)	24 (5%)	12 (2%)	15 (3%)
	Total central dense (CC3)	0	0	3 (1%)	1 (<1%)
	Phthisis (CC4)	0	0	0	0
Tarsal conjunctiva inflammation					
	None (P0)	7 (1%)	9 (2%)	39 (8%)	48 (10%)
	Mild (P1)	78 (16%)	87 (17%)	235 (48%)	224 (46%)
	Moderate (P2)	278 (55%)	288 (58%)	197 (40%)	189 (39%)
	Severe (P3)	138 (28%)	115 (23%)	23 (5%)	27 (6%)
Tarsal conjunctival scarring at baseline					
	None (C0)	0	0	..	..
	Mild (C1)	15 (3%)	22 (4%)	..	..
	Moderate (C2)	405 (81%)	405 (81%)	..	..
	Severe (C3)	81 (16%)	72 (14%)	..	..
Tarsal conjunctival scarring at 12 months					
	None (SC0)	..	..	0	0
	Surgical line only (SC1)	..	..	0	0
	Scattered scar (SC2)	..	..	2 (<1%)	4 (1%)
	Widespread scar (SC3)	..	..	360 (73%)	321 (66%)
	Widespread scar and distortion from surgery (SC4)	..	..	121 (24%)	155 (32%)
	Widespread scar and distortion from scar (SC5)	..	..	11 (2%)	8 (2%)

Data are n (%), or mean (SD). logMAR=logarithm of the minimum angle of resolution. CF=counting fingers. HM=hand motion. PL=perception of light. NPL=no perception of light.

* Excluding participants with no eyelashes touching the eyeball.

By 12 months, postoperative trichiasis had developed in 58 (12%) of 498 participants in the doxycycline group and 61 (12%) of 501 participants in the placebo group (adjusted OR 0·91, 95% CI 0·61 to 1·34, p=0·63). The risk difference for postoperative trichiasis between the doxycycline and placebo groups was −0·5% (95% CI −4·5% to 3·5%). When testing for effect modification using prespecified variables, results indicated that the intervention effect was modified by sex (p_interaction_=0·02); however, the stratified estimates indicated that doxycycline did not affect the risk of postoperative trichiasis in women (OR 0·67, 95% CI 0·42–1·07, p=0·10) or men (OR 1·90, 0·88–4·11, p=0·10).

A per-protocol analysis of the primary outcome, adjusted for surgeon and triachiasis severity, found no difference in the cumulative risk by treatment group: 47 (12%) of 392 participants in the doxycycline group and 51 (12%) of 423 participants in the placebo group developed postoperative trichiasis by 12 months (OR 1·00, 95% CI 0·65–1·53, p=0·99). Evidence of effect modification by sex was found (p_interaction_=0·01), and men in the doxycycline group were found to have an increased risk of postoperative trichiasis compared with men in the placebo group, but this association was weak (OR 2·31, 95% CI 0·99–5·40, p=0·054). However, no significant differences in risk of postoperative triachisis were identified between women in the doxycycline and placebo groups (OR 0·66, 95% CI 0·39–1·11, p=0·12).

The proportion of participants with postoperative trichiasis was similar across the two groups at the 10 day follow-up, and 1, 6, and 12 month follow-up assessments (table 2). The cumulative risks of postoperative trichiasis in patients with major trichiasis and severe entropion were similar between the groups. No significant differences in the number or location of postoperative trichiasis eyelashes were identified between the two treatment groups at 12 months. The proportion of participants with granuloma by 12 months and eyelid contour abnormalities at 10 days, 1 month, 6 months and 12 months was similar between treatment groups (table 2). No significant differences in visual acuity (p=0·88), corneal opacity (p=0·71), or entropion (p=0·39) were identified between the treatment groups at 12 months.

**Table 2:** Primary and secondary clinical outcomes and changes in clinical phenotype

		**Placebo**	**Doxycycline**	**OR (95% CI)**	**p value**
Cumulative proportion of patients with postoperative trichiasis*		61/501 (12%)	58/498 (12%)	0·91 (0·61–1·34)	0·63
Postoperative trachomatous trichiasis at 10 days*		3/499 (1%)	1/497 (<1%)	0·33 (0·03–3·2)	0·34
Postoperative trachomatous trichiasis at 1 month*		9/500 (2%)	5/492 (1%)	0·55 (0·18–1·7)	0·29
Postoperative trachomatous trichiasis at 6 months*		32/493 (6%)	29/481 (6%)	0·86 (0·50–1·46)	0·57
Postoperative trachomatous trichiasis at 12 months*		45/494 (9%)	47/492 (10%)	1·01 (0·65–1·57)	0·97
Cumulative postoperative trichiasis by baseline trichiasis severity†					
	Minor trachomatous trichiasis	20/279 (7%)	23/244 (9%)	1·41 (0·74–2·66)	0·29
	Major trachomatous trichiasis	41/222 (18%)	35/254 (14%)	0·72 (0·43–1·19)	0·20
Cumulative postoperative trichiasis by baseline entropion severity†					
	None or mild	0	4/27 (15%)	..	..
	Moderate	48/415 (12%)	46/408 (11%)	0·97 (0·63–1·51)	0·91
	Severe	13/64 (20%)	8/63 (13%)	0·63 (0·24–1·67)	0·35
Number of postoperative trichiasis eyelashes at 12 months (mean, SD)‡		2·5 (3·9)	1·9 (1·4)	0·85§ (0·58–1·25)	0·42
Location of postoperative trichiasis eyelashes at 12 months¶					
	None (epilating)	4/44 (9%)	3/47 (6%)	0·59‖ (0·11–3·21)	0·57
	Corneal	28/44 (64%)	31/47 (66%)	1	..
	Corneal and peripheral	5/44 (11%)	3/47 (6%)	0·37‖ (0·07–1·94)	0·24
	Peripheral	7/44 (16%)	10/47 (21%)	0·92‖ (0·27–3·18)	0·90
Postoperative trichiasis by surgeon**					
	1	7/99 (7%)	8/102 (8%)	1·15 (0·40–3·31)	0·80
	2	17/99 (17%)	13/97 (13%)	0·79 (0·35–1·78)	0·57
	3	5/101 (5%)	11/99 (11%)	2·21 (0·73–6·70)	0·16
	4	15/99 (15%)	16/98 (16%)	1·00 (0·45–2·19)	0·10
	5††	1/2 (50%)	1/2 (50%)	1·00 (0·02–50·4)	1·00
	6	16/101 (16%)	9/100 (9%)	0·52 (0·22–1·23)	0·14
Eyelid contour abnormalities at 1 month¶					
	None (base outcome)	321/500 (64%)	311/492 (63%)	1	..
	Clinically non-significant (mild)	107/500 (21%)	108/492 (22%)	1·04‖ (0·76–1·42)	0·80
	Clinically significant (moderate and severe)	72/500 (14%)	73/492 (15%)	1·03‖ (0·72–1·48)	0·87
Eyelid contour abnormalities at 6 months¶					
	None (base outcome)	411/493 (83%)	393/481 (82%)	1	..
	Clinically non-significant (mild)	46/493 (9%)	55/481 (11%)	1·25‖ (0·83–1·90)	0·76
	Clinically significant (moderate and severe)	36/493 (7%)	33/481 (7%)	0·94‖ (0·57–1·54)	0·81
Eyelid contour abnormality at 12 months¶					
	None (base outcome)	439/494 (89%)	422/488 (86%)	1	..
	Clinically non-significant (mild)	35/494 (7%)	37/488 (8%)	1·08‖ (0·66–1·75)	0·76
	Clinically significant (moderate and severe)	20/494 (4%)	29/488 (6%)	1·47‖ (0·81–2·65)	0·20
Granuloma by 12 months*		58/501 (12%)	51/498 (10%)	0·87 (0·58–1·30)	0·49
Sign of infection at 7–14 days‡‡		41/499 (9%)	39/497 (8%)	0·94 (0·59–1·49)	0·79
Tarsal conjunctiva inflammation at 1 month§§		..	..	0·88 (0·69–1·11)	0·23
	None (P0)	4/500 (1%)	3/492 (1%)	..	..
	Mild (P1)	196/500 (39%)	210/492 (43%)	..	..
	Moderate (P2)	240/500 (48%)	228/492 (46%)	..	..
	Severe (P3)	60/500 (12%)	51/492 (11%)	..	..
Tarsal conjunctiva inflammation at 6 months§§		..	..	0·84 (0·64–1·10)	0·21
	None (P0)	1/493 (<1%)	4/481 (1%)	..	..
	Mild (P1)	332/493 (67%)	332/481 (69%)	..	..
	Moderate (P2)	143/493 (29%)	134/481 (28%)	..	..
	Severe (P3)	17/493 (3%)	11/481 (2%)	..	..
Tarsal conjunctiva scarring at 6 months§§		..	..	1·22 (0·85–1·77)	0·28
	None (SC0)	..	..	..	..
	Surgical line only (SC1)	..	..	..	..
	Scattered scar (SC2)	4/493 (1%)	3/481 (1%)	..	..
	Widespread scar (SC3)	431/493 (87%)	409/481 (85%)	..	..
	Widespread scar and distortion from surgery (SC4)	56/493 (11%)	66/481 (14%)	..	..
	Widespread scar and distortion from scar (SC5)	2/493 (<1%)	3/481 (1%)	..	..
Adverse events¶¶		6/501 (1%)	18/499 (4%)	3·09 (1·21–7·84)	0·02

Data are n/N (%), unless otherwise stated. OR=odds ratio.

* Analysis done using logistic regression adjusted for surgeon and trichiasis severity at baseline.

† Analysis done using logistic regression adjusted for surgeon to see the effect of the two surgical procedures on cumulative postoperative trichiasis (by 12 months) across baseline trichiasis and entropion severity level.

‡ Analysis done using negative binomial regression adjusted for surgeon and trichiasis severity at baseline.

§ Incidence rate ratio.

¶ Analysis done by multinomial logistic regression adjusted for surgeon and trichiasis severity at baseline.

‖ Relative risk ratio.

** Analysis done by logistic regression adjusted for trichiasis severity at baseline.

†† Surgeon 5 moved to another region of Ethiopia immediately after the start of the trial.

‡‡ All patients had mild infections, which improved without further treatment.

§§ Analysis done by ordinal logistic regression adjusted for surgeon and trichiasis severity at baseline.

¶¶ Analysis done by logistic regression.

Eyelid contour abnormalities were more common at the 1 month follow-up than at the 6 month and 12 month follow-up assessments in both groups (table 2). Many participants with eyelid contour abnormalities at 1 month had statistically significant regression by 6 months (mild to none, 158 [77%] of 206 participants; moderate to mild or none, 86 [70%] of 122 participants; severe to mild or none, ten [48%] of 21 participants; sign test p<0·0001) and by 12 months (moderate to mild or none, 97 [79%] of 123 participants; severe to mild or none, 11 [52%] of 21 participants, sign test p<0·0001, table 3). Similar changes were also observed between the 6 month and 12 month follow-up assessments (mild to none, 68 [67%] of 101 participants; moderate to mild or none, 28 [44%] of 63 participants, sign test p<0·0001). However, no significant differences were identified between the doxycycline group and the placebo group (table 3). Of the 621 participants who did not have eyelid contour abnormalities at 1 month, 27 (4%) developed eyelid contour abnormalities by the 6 month follow-up and eight (1%) by the 12 month follow-up. Of the 797 participants without eyelid contour abnormalities at 6 months, 12 (1·5%) had developed eyelid contour abnormalities by 12 months. Of the 143 participants with clinically significant eyelid contour abnormalities at 1 month, 47 (33%) still had clinically significant eyelid contour abnormalities at 6 months and 36 (25%) at 12 months (table 3)**.**

**Table 3:** Changes in eyelid contour abnormality grades between follow-up assessments

	**1 month to 6 months**			**1 month to 12 months**			**6 months to 12 months**		
	Placebo (%)	Doxycycline	RRR* (95% CI)	Placebo	Doxycycline	RRR (95% CI)	Placebo	Doxycycline	RRR (95% CI)
**No eyelid contour abnormalities**									
None	301/317 (95%)	293/304 (96%)	1 (ref)	312/317 (98%)	301/304 (99%)	1 (ref)	402/408 (99%)	383/389 (98%)	1 (ref)
None to mild	14/317 (4%)	11/304 (4%)	0·81 (0·36–1·81)	3/317 (1%)	1/304 (<1%)	0·34 (0·04–3·34)	6/408 (1%)	5/389 (1%)	0·87 (0·26–2·89)
None to moderate	2/317 (1%)	0	..	1/317 (<1%)	2/304 (1%)	2·07 (0·19–23·0)	0	1/389 (<1%)	..
None to severe	0	0	..	1/317 (<1%)	0	..	0	0	..
**Mild eyelid contour abnormalities**									
Mild to none	84/104 (81%)	74/102 (73%)	0·42 (0·18–0·98)	0	0	..	36/46 (78%)	32/55 (58%)	0·44 (0·15–1·13)
Remain mild	9/104 (9%)	19/102 (19%)	1 (ref)	105/106 (98%)	101/106 (96%)	1 (ref)	9/46 (20%)	18/55 (32·7%)	1 (ref)
Mild to moderate	11/104 (11%)	9/102 (9%)	0·39 (0·12–1·27)	2/106 (2%)	5/106 (5%)	2·60 (0·49–13·7)	0	4/55 (7%)	2·00 (0·19–20·6)
Mild to severe	0	0	..	0	2/106 (2%)	..	0	1/55 (2%)	..
**Moderate eyelid contour abnormalities**									
Moderate to none	25/64 (39%)	19/58 (33%)	0·86 (0·34–2·15)	32/63 (51%)	30/60 (50%)	0·60 (0·20–1·74)	6/34 (18%)	2/29 (7%)	0·25 (0·04–1·46)
Moderate to mild	22/64 (34%)	20/58 (34%)	1·03 (0·41–2·59)	21/63 (33%)	14/60 (23%)	0·42 (0·13–1·36)	12/34 (35%)	8/29 (28%)	0·50 (0·16–1·60)
Remain moderate	17/64 (27%)	15/58 (26%)	1 (ref)	7/63 (11%)	11/60 (18%)	1 (ref)	12/34 (35%)	16/29 (55%)	1 (ref)
Moderate to severe	0	4/58 (7%)	..	3/63 (5%)	5/60 (8%)	1·06 (0·19–5·90)	4/34 (12%)	3/29 (10%)	0·56 (0·10–3·00)
**Severe eyelid contour abnormalities**									
Severe to none	1/8 (13%)	3/13 (23%)	2·40 (0·17–32·9)	1/8 (13%)	4/13 (31%)	6·67 (0·49–91·3)	0	0	..
Severe to mild	1/8 (13%)	5/13 (38%)	4·00 (0·32–49·6)	1/8 (13%)	5/13 (38%)	8·33 (0·63–110)	0	0	..
Severe to moderate	4/8 (50%)	5/13 (38%)	1 (ref)	5/8 (63%)	3/13 (23%)	1 (ref)	1/2 (50%)	0	..
Remain severe	2/8 (25%)	0	..	1/8 (13%)	1/13 (8%)	1·67 (0·07–37·7)	1/2 (50%)	4/4 (100%)	..
**Clinically significant eyelid contour abnormalities**									
Clinically significant to none	26/72 (36%)	22/71 (31%)	0·81 (0·36–1·81)	33/71 (46%)	34/73 (47%)	0·82 (0·36–1·86)	6/36 (17%)	2/33 (6%)	0·26 (0·05–1·45)
Clinically significant to mild	23/72 (32%)	25/71 (35%)	1·04 (0·47–2·33)	22/71 (31%)	19/73 (26%)	0·69 (0·28–1·70)	12/36 (33%)	8/33 (24%)	0·52 (0·18–1·55)
Remain clinically significant	23/72 (32%)	24/71 (34%)	1 (ref)	16/71 (23%)	20/73 (27%)	1 (ref)	18/36 (50%)	23/33 (70%)	1 (ref)

Data are n/N (%). Changes in eyelid contour abnormalities were assessed in patients between two follow-up assessments, and thus the number of patients with each type of eyelid contour abnormality does not always equal the number presented in table 2, which reported the number of patients with eyelid contour abnormalities at a single timepoint. RRR=relative risk ratio.

* Multinomial logistic regression.

Mild adverse events were reported by 18 (4%) of 498 participants in the doxycycline group and six (1%) of 501 participants in the placebo group (OR 3·09, 95% CI 1·21–7·84, p=0·02; table 2). The five most frequent adverse events were gastritis symptoms (n=10 doxycycline group; n=1 placebo group), constipation (n=4 doxycycline group; n=2 placebo group), diarrhoea (n=4 doxycycline group; n=1 placebo group), generalised weakness (n=3 doxycycline group; n=1 placebo group), and anorexia (n=3 doxycycline group; n=1 placebo group). No treatment-related serious adverse events were reported.

We did univariable and multivariable analyses of factors associated with postoperative trichiasis by 12 months in all trial participants (table 4), which showed that older age, major trichiasis at baseline, peripheral eyelashes at baseline, surgeon, and postoperative under-correction independently predicted postoperative trichiasis, whereas eyelid contour abnormalities at 12 months were associated with a significantly lower risk of postoperative trichiasis.

**Table 4:** Univariable and multivariable associations between clinical and demographic factors and postoperative trichiasis by 12 months

		**Patients with postoperative trachomatous trichiasis, n/N (%)**	**Univariable analysis**		**Multivariable analysis**	
			OR (95% CI)	p value	OR (95% CI)	p value
Sex			0·97 (0·63–1·48)	0·90	Not included	Not included
	Women	84/710 (12%)	..	..	..	..
	Men	35/289 (12%)	..	..	..	..
Age, years			1·10 (0·96–1·27)	0·17*	1·21 (1·04–1·41)	0·01*
	18–29	5/77 (6%)	..	..	..	..
	30–39	12/124 (10%)	..	..	..	..
	40–49	25/206 (12%)	..	..	..	..
	50–59	35/262 (13%)	..	..	..	..
	60–69	32/238 (13%)	..	..	..	..
	≥70	10/92 (11%)	..	..	..	..
Treatment group			0·95 (0·65–1·39)	0·80	0·91 (0·60–1·37)	0·91
	Placebo	61/501 (12%)	..	..	..	..
	Doxycycline	58/498 (12%)	..	..	..	..
Trichiasis severity at baseline			2·12 (1·43–3·15)	0·0002	2·20 (1·44–3·36)	0·0003
	Minor	43/523 (8%)	..	..	..	..
	Major	76/476 (16%)	..	..	..	..
Entropion grade at baseline			1·52 (0·97–2·38)	0·07*	Not included	Not included
	None or mild	4/49 (8%)	..	..	..	..
	Moderate	94/823 (11%)	..	..	..	..
	Severe	21/127 (17%)	..	..	..	..
Eyelash location at baseline						
	Epilating	8/59 (14%)	1·30 (0·60–2·83)	0·51	1·56 (0·69–3·54)	0·29
	Corneal	86/800 (11%)	1 (ref)	..	1 (ref)	..
	Corneal and peripheral	22/130 (17%)	1·69 (1·01–2·82)	0·04	1·47 (0·85–2·56)	0·17
	Peripheral	3/10 (30%)	3·56 (0·90–14·0)	0·07	6·00 (1·33–26·92)	0·02
Papillary grade at baseline			0·97 (0·74–1·28)	0·83*	Not included	Not included
	None	2/16 (13%)	..	..	..	..
	Mild	21/165 (13%)	..	..	..	..
	Moderate	66/566 (12%)	..	..	..	..
	Severe	30/252 (12%)	..	..	..	..
Conjunctival scarring grade at baseline			1·07 (0·68–1·69)	0·76*	Not included	Not included
	Mild	6/37 (16%)	..	..	..	..
	Moderate	92/810 (11%)	..	..	..	..
	Severe	21/152 (14%)	..	..	..	..
Surgeon						
	1	15/201 (7%)	1 (ref)	..	1 (ref)	..
	2	30/196 (15%)	2·24 (1·16–4·31)	0·02	2·36 (1·19–4·67)	0·01
	3	16/200 (8%)	1·08 (0·52–2·24)	0·84	1·29 (0·60–2·73)	0·51
	4	31/197 (16%)	2·32 (1·21–4·44)	0·01	2·02 (1·02–4·00)	0·04
	5	2/4 (50%)	12·4 (1·63–94·3)	0·02	19·51 (1·99–191·5)	0·01
	6	25/201 (12%)	1·76 (0·90–3·45)	0·10	1·87 (0·92–3·78)	0·08
Postoperative central correction at 12 months						
	Corrected	94/881 (11%)	1 (ref)	..	1 (ref)	..
	Over-corrected	2/50 (4%)	0·35 (0·08–1·46)	0·15	1·22 (0·19–7·96)	0·83
	Under-corrected	23/51 (45%)	6·9 (3·81–12·4)	<0·0001	6·97 (3·67–13·2)	<0·0001
Papillary grade at 12 months			1·17 (0·90–1·53)	0·24*	Not included	Not included
	None	3/87 (3%)	..	..	..	..
	Mild	63/459 (14%)	..	..	..	..
	Moderate	46/386 (12%)	..	..	..	..
	Severe	7/50 (14%)	..	..	..	..
Eyelid contour abnormalities at 12 months			0·22 (0·08–0·61)	0·004	0·18 (0·05–0·68)	0·01
	No	57/371 (15%)	..	..	..	..
	Yes	6/120 (5%)	..	..	..	..

Analysis was done by logistic regression. Age (known risk factor for postoperative trichiasis) was included in the multivariable model regardless of significance in univariable analysis. Likelihood ratio test was done to determine the variables for inclusion in the final multivariable model. OR=odds ratio.

* p value for trend.

The presence of eyelid contour abnormalities at 12 months, irrespective of treatment group, was associated with increasing age (OR 1·46, 95% CI 1·25–1·71, p<0·0001), major trichiasis at baseline (minor trichiasis, 51 [10%] of 516 patients *vs* major trichiasis, 70 [15%] of 466 patients; OR 1·47, 0·99–2·18, p=0·054), and surgeon (surgeon 4, 16 [8%] of 196 patients *vs* surgeon 6, 36 [19%] of 192 patients; OR 2·65, 1·40–5·01, p=0·003). All other variables assessed were not associated with the presence of eyelid contour abnormalites (data not shown). The prevalence of granuloma during the 12 month period was associated with surgeon (surgeon 4, 13 [7%] of 197 patients *vs* surgeon 2, 31 [16%] of 196 patients; OR 2·59, 1·30–5·16, p=0·007), and eyelid contour abnormalities (no eyelid contour abnormalities, 81 [9%] of 861 patients *vs* eyelid contour abnormalities, 27 [22%] of 121 patients; OR 3·11, 1·87–5·19, p<0·0001), whereas older age seemed to have a protective effect (OR 0·88, 0·75–1·02, p=0·09). All other variables assessed were not associated with the prevalence of granuloma (data not shown).

## Discussion

We tested the hypothesis that doxycycline could reduce the risk of postoperative trichiasis by reducing MMP and pro-inflammatory cytokine activity, thereby reducing inflammation and fibrosis during the initial wound healing stage. However, in this trial, we found no evidence to support this theory, with similar surgical outcomes observed between the doxycycline and placebo groups. To the best of our knowledge, this is the first study to have investigated the effect of doxycycline on surgical outcomes after trichiasis surgery. Although, fewer people in the doxycycline group adhered to study treatment than did those in the placebo group, per-protocol analysis found no difference in the risk of postoperative trichiasis between groups, when the analysis was restricted to participants who reported adherence at 1 month.

The rationale for this trial was that persistent conjunctival inflammation and progressive scarring after trichiasis surgery might contribute to the development of postoperative trichiasis. Several studies^10^, ^12^, ^13^, ^17^, ^44^, ^45^ have found severe conjunctival inflammation before and after surgery is associated with postoperative trichiasis. Preoperatively, clinically visible conjunctival inflammation and scarring are associated with increased expression of pro-inflammatory cytokines (interleukin-1β, CXCL5, and S100A7) and MMPs (MMP-7, MMP-9 and MMP-12).^16^, ^21^ Similarly, increased expression of these factors has also been reported in patients following trichiasis surgery.^23^ MMPs and pro-inflammatory cytokines are integral to wound healing and tissue remodelling processes, thus the excessive inflammation and profibrotic reaction in response to trichiasis surgery could plausibly result in poorer patient outcomes after surgery. Doxycycline has well characterised effects on these processes, suggesting potential benefit.

No differences in the severity of tarsal conjunctival scarring were found between the treatment groups at 6 months or 12 months after surgery. Tarsal conjunctival scarring progresses gradually, increases with age, and is particularly associated with conjunctival inflammation.^20^ It is possible that in some patients, scarring continues to progress after trichiasis surgery, contributing to postoperative trichiasis. The results of our study indicate that 1 month of oral doxycycline treatment does not alter the degree of postoperative conjunctival scarring, whether this is associated with the initial wound healing process or longer-term chronic progression. Similarly, we did not observe a difference between treatment groups in the extent of clinically visible conjunctival inflammation 1 month after surgery.

The risk of postoperative trichiasis 1 year after PLTR surgery was relatively low (12% in both groups). These results were similar to those previously reported for PLTR surgery (13%),^7^ suggesting consistent performance of this procedure in this context. This risk was lower than anticipated in the power calculation. However, our study had sufficient power to detect a halving of the postoperative trichiasis risk from 12% to 6%. Some evidence indicated that the intervention effect might be modified by sex; however, stratum-specific estimates did not support this finding. However, because the study was not powered to detect a difference within these strata we cannot make robust conclusions about the absence of an effect. In this study, the main determinants of postoperative trichiasis were preoperative trichiasis severity (major trachomatous trichiasis), peripheral trichiasis eyelash location, intersurgeon variability, and patient age, which have consistently been reported in previous trials.^6^, ^46^

The risk of clinically significant (moderate and severe) eyelid contour abnormalities after trichiasis surgery was similar between treatment groups throughout the 12 month follow-up. The risk was relatively high at 1 month (14% in placebo group *vs* 15% in doxycycline group), but declined to 7% in the placebo group and 7% in the doxycycline group by 6 months and to 4% in the placebo group and 6% in the doxycycline group by 12 months. Most participants who had clinically significant eyelid contour abnormalities at 1 month had improvement by 6 months and 12 months: only 33% of participants at 6 months and 25% of participants at 12 months still had clinically significant eyelid contour abnormalities. The gradual improvement in appearance of eyelid contour abnormalities is presumably due to ongoing changes within the eyelid tissues, with reshaping of scar tissue, which has implications for both patients and trachoma control programmes. Poor cosmetic outcome is sometimes cited as a reason to decline surgery, so it is encouraging that eyelid contour abnormalities can improve. Additionally, these results suggest that for all but the most severe eyelid contour abnormalities, in which clinically significant incomplete eyelid closure (lagophthalmos) might occur, repeat eyelid surgery might not be required since improvement occurs in most patients. Long-term longitudinal studies are required to determine the proportion of patients who have surgery but remain with disfiguring eyelid contour abnormalities and how these should be managed.

The factors associated with eyelid contour abnormalities were similar to those for postoperative trichiasis—ie, preoperative trichiasis severity (major trachomatous trichiasis), intersurgeon variability, and patient age. The only modifiable factor was surgeon skill, indicating the need for robust training programmes and ongoing supervision. Patients with severe trichiasis should be managed by the most experienced surgeons wherever possible.

Although doxycycline had no effect on the outcome of trichiasis surgery in this trial, the wider ophthalmic literature^26^, ^27^, ^28^, ^29^, ^30^, ^31^, ^32^, ^33^, ^34^ suggests that the drug might be beneficial in a number of different clinical situations. In ophthalmic practice, the drug is used to help promote healing of recurrent corneal erosions, possibly via anti-MMP-9 activity.^47^ Oral doxycycline is reported to improve chronic blepharitis, possibly in association with anti-MMP-9 activity.^48^ Two case reports^29^ of pseudomonas corneal melting suggest oral doxycycline helps to stabilise corneal breakdown via the inhibition of MMPs, and is widely used in clinical practice for this reason. Oral doxycycline was reported to be effective in inhibiting corneal neovascularisation and improved epithelia cell healing in rat corneas exposed to alkali, and is widely used in clinical practice for the treatment of alkali injuries.^49^

The use of doxycycline in the treatment of systemic conditions has had mixed results. The drug is licensed for use in the treatment of periodontitis; several placebo-controlled randomised clinical trials^50^ have demonstrated that sub-antimicrobial doses can suppress MMP-mediated degradation of connective tissue in the periodontal structures. Clinical trials^31^ have also shown that patients with rosacea respond to anti-inflammatory doses of oral doxycycline. Several studies^51^ have examined whether the enlargement of abdominal aortic aneurysms can be slowed by oral doxycycline, mediated through opposing MMP-9 activity, but no evidence has suggested that it is effective for this indication. Doxycycline has also been proposed as a treatment in pulmonary fibrosis, although convincing trial data are not available at present.

In this study, treatment-associated adverse events were uncommon and generally mild. Adverse events were more frequently reported with doxycycline than placebo, which might explain why significantly fewer people in the doxycycline group were compliant when compared with the placebo group. The risk and type of advese events reported were similar to those reported in a large community-based trial^35^ of doxycycline (100 mg per day for 6 weeks) for onchocerciasis in Cameroon, in which 3% of the trial participants reported nausea, vomiting, diarrhoea, and tiredness.

A potential limitation of this trial is the risk of unmasking when examiners counted the number of capsules left in the randomisation box to collect adherence data. However, we consider this highly unlikely since the two treatment capsules were identical.

Overall, doxycycline was found to have no effect on the proportion of participants who developed postoperative trichiasis or other unfavourable outcomes, and therefore is not indicated for this purpose. Preoperative disease characteristics, patient age, and surgeon skill seem to be the main determinants of outcome following trichiasis surgery. Trachoma control programmes and their partners should continue to make efforts to improve surgical training and supervision.
